# Personality traits, sex and food abundance shape space use in an arboreal mammal

**DOI:** 10.1007/s00442-021-04901-2

**Published:** 2021-04-01

**Authors:** Lucas A. Wauters, Maria Vittoria Mazzamuto, Francesca Santicchia, Adriano Martinoli, Damiano G. Preatoni, Peter W. W. Lurz, Sandro Bertolino, Claudia Romeo

**Affiliations:** 1 Environment Analysis and Management Unit, Guido Tosi Research Group, Department of Theoretical and Applied Sciences, Università degli Studi dell’Insubria, via J.H. Dunant, 3, 21100 Varese, Italy; 2 Department of Biology, University of Antwerp, Wilrijk, Belgium; 3 School of Natural Resources and the Environment, University of Arizona, Tucson, USA; 4 The Royal (Dick) School of Veterinary Studies and the Roslin Institute, University of Edinburgh, Roslin, UK; 5 Department of Life Sciences and Systems Biology, Università degli Studi di Torino, Turin, Italy; 6 Department of Veterinary Medicine, Università degli Studi di Milano, Milan, Italy

**Keywords:** Core-area, Home-range size, *Sciurus vulgaris*, Seed-crop size, Trappability

## Abstract

Animal space use is affected by spatio-temporal variation in food availability and/or population density and varies among individuals. This inter-individual variation in spacing behaviour can be further influenced by sex, body condition, social dominance, and by the animal’s personality. We used capture-mark-recapture and radio-tracking to examine the relationship between space use and personality in Eurasian red squirrels (*Sciurus vulgaris*) in three conifer forests in the Italian Alps. We further explored to what extent this was influenced by changes in food abundance and/or population density. Measures of an individual’s trappability and trap diversity had high repeatability and were used in a Principal Component Analysis to obtain a single personality score representing a boldness-exploration tendency. Males increased home-range size with low food abundance and low female density, independent of their personality. However, bolder males used larger core-areas that overlapped less with other males than shy ones, suggesting different resource (food, partners) utilization strategies among personality types. For females, space use-personality relationships varied with food abundance, and bolder females used larger home ranges than shy ones at low female density, but the trend was opposite at high female density. Females’ intrasexual core-area overlap was negatively related to body mass, with no effect of personality. We conclude that relationships between personality traits and space use in free-ranging squirrels varied with sex, and were further influenced by spatio-temporal fluctuations in food availability. Moreover, different personality types (bold-explorative vs. shy) seemed to adopt different space-use strategies to increase access to food and/or partners.

**Supplementary Information:**

The online version contains supplementary material available at 10.1007/s00442-021-04901-2.

## Introduction

Animals need to travel in search of resources such as food, refuge, nesting site or mates, making movement and space use key aspects of their behaviour, survival and reproductive success (Burt 1943; Moorcroft 2012). Spatial dynamics, therefore, have important consequences in ecological issues like gene flow (through dispersal), species distribution, population and community dynamics, and, therefore, are relevant for conservation (Nathan et al. 2008; Cote et al. 2010; Kays et al. 2015). A species’ movement ecology determines how individuals will interact with their conspecifics, with other species and their environment, ultimately shaping the spatial structure of communities and ecosystems (van Beest et al. 2011; Tamburello et al. 2015).

Animal movement patterns can vary between and within species, and the size, shape or structure of the space utilised can be affected by both extrinsic and intrinsic factors, which have been widely studied for many species (McLoughlin and Ferguson 2000; Tucker et al. 2014). At the population level, extrinsic factors such as habitat quality and heterogeneity are relevant in shaping animal space use (Wauters et al. 2001). For example, abundant food resources often lead to smaller home-range size (e.g. Šálek et al. 2015), which in turn may lead to changes in the intensity of inter- and intraspecific interactions and, sometimes, social organisation (Joshi et al. 1995; Jetz et al. 2004). Space use has been widely demonstrated to vary also within populations and this inter-individual variation in animal movement and home-range size has led to the concept of individual niche specialization (Schirmer et al. 2019). For instance, an animal’s home range can be affected by intrinsic factors such as its age, sex or body size (e.g. Wauters and Dhondt 1992; Lurz et al. 2000; Frafjord 2016). However, a substantial part of this intraspecific, individual variation in home-range size and movement patterns remains unexplained (van Overveld and Matthysen 2010; Moorcroft 2012; Cote et al. 2014). A growing number of studies has acknowledged that consistent individual variation in space use is related to differences in personality (Réale et al. 2010; Cote et al. 2014; Spiegel et al. 2017; Schirmer et al. 2019), defined as among-individual differences in behaviour that persist through time and under different ecological contexts (Réale et al. 2007; Biro and Stamps 2008; Carter et al. 2013).

In many species, personality traits are measured using the movement response of an individual to stimuli. An exploratory individual is one who, exposed to a new environment and/or object (e.g. open field test), will explore longer and/or faster; a bold individual is one who will move more into riskier environments (more implicit concept of movement) (Walsh and Cummins 1976; Spiegel et al. 2017; Mazzamuto et al. 2019). Hence, since personality traits affect how individuals perceive and interact with their environment, they are likely to influence the way individuals adapt their space use to fluctuating environmental conditions (Haughland and Larsen 2004). Such relationships between personality and space use, with differences in personality affecting movement, settlement and home-range size, have been documented for several vertebrate species (Spiegel et al. 2015, 2017; Merrick and Koprowski 2017; Cooper et al. 2017; Villegas‐Ríos et al. 2018; Schirmer et al. 2019; Wat et al. 2019).

Moreover, since the existence of personality types implies that animals may respond differently to constraints, the effects of variation in personality on an individual’s space use should be more pronounced when it experiences marked changes in environmental conditions, such as reduced resource availability and/or increased population density (Sih et al. 2018). Although this is theoretically well established, few studies have considered the potential interactions between personality, the animal’s sex and fluctuating environmental conditions in affecting spacing behaviour. We used multi-year and multi-site data, covering a wide range in fluctuating environmental conditions (food availability and sex-specific population density), to explore how the animal’s sex and changes in extrinsic factors interact with the complex relationships between space use and personality, using an arboreal rodent as study species.

Researchers who want to study personality-dependent spatial ecology in wild animals can adopt two approaches. One is to use animal spatial data to assess whether repeatable, between-individual differences in space use exist (Boon et al. 2008; Harrison et al. 2015; Hertel et al. 2019). However, individual differences in personality and behavioural plasticity to environmental conditions both contribute to observed behavioural differences. Caution must therefore be exercised when trying to disentangle these factors (Hertel et al. 2020). The second approach is the use of a two-step method where the animal is first captured and tested in a novel, standard, and controlled environment to determine behavioural responses. The animal is then released to relate the test to natural behaviours measured in the wild (e.g. Merrick and Koprowski 2017). This second approach is not always applicable, because of the difficulties related to creating a standard, repeatable controlled test in the wild; and/or when large species are involved that are difficult to manage. Moreover, interpretation of the correlations between behaviours displayed in an artificial environment and in the wild must be made carefully (Niemelä and Dingemanse 2014).

In this study, we aimed to investigate how the animal’s sex and differences in the environmental context (variation in food abundance, population density) can shape the relationship between space use of adult Eurasian red squirrels (*Sciurus vulgaris*) and their personality along a bold, more explorative—shy, less explorative continuum. Earlier studies showed that spacing behaviour differs between male and female squirrels, and that red squirrels tend to increase their home ranges in response to poor food availability and/or when densities are low (Wauters and Dhondt 1992; Lurz et al. 2000; Romeo et al. 2010; Di Pierro et al. 2011). The novelty in this study is that we incorporate temporal fluctuations in food availability and population density to explore how an animal’s personality affects individual variation in space use within a given habitat (or population), and whether these associations vary with the squirrel’s sex.

We radio-tracked squirrels to determine home-range and core-area (i.e. the most intensively used part of the entire home range) size and inter-individual core-area overlap (Wauters et al. 2007; Di Pierro et al. 2008). We also calculated the core-area/home-range ratio as a proxy of home range structure. For each of these radio-tracked individuals, we used a capture-mark-recapture derived trappability index (as measure of boldness) and trap diversity index (as measure of exploration) (Boyer et al. 2010; Santicchia et al. 2018, 2020). We predict that: (1) bolder, more explorative individuals of both sexes will have larger home ranges than shy-less explorative ones, due to frequent excursions further away from the core-area and to react faster to changing environmental situations; moreover, they might also have larger core-areas to increase the areas of intensive foraging; (2) bolder, more explorative individuals will have higher core-area/home range ratio, meaning that they will intensively use a larger proportion of their home range; (3) as a consequence of predictions 1 and 2, core-area overlap of bolder, more explorative squirrels should be higher than for shyer, less explorative ones. Finally, since extrinsic factors such as food availability and density of conspecifics are known to greatly affect space use of squirrels (Wauters and Dhondt 1992; Wauters et al. 2005; Romeo et al. 2010; Di Pierro et al. 2011), we expect that the relationships between personality traits and spatial behaviour of squirrels could be enhanced by fluctuating environmental and/or social conditions. In particular, we predict that (4) while male home ranges will vary inversely in size with food availability and with the density of females, the personality-space use relationship in females will be mainly affected by food availability to sustain energy requirements of lactation and thus enhance reproductive success (female space use is more strongly food-dependent than for males; Wauters and Dhondt 1992, 1995; Di Pierro et al. 2011).

## Materials and methods

### Study species

In the Alps, the Eurasian red squirrel (henceforth referred to as red squirrel) mostly feeds on conifer seeds in the tree canopy from late summer to early next spring, and, in some forest types, recovers scatter-hoarded cones in late spring when no new cones are available in the trees. Some buds, shoots, and flowers of conifers, fungi and berries can also be consumed in late spring and summer (Romeo et al. 2010; Di Pierro et al. 2011).

Red squirrels have overlapping home ranges, with more intensively used core-areas. Home-range and core-area size differ markedly among habitat types, based on overall habitat quality (food resource availability) and squirrel density, and home-range size can fluctuate seasonally (Wauters and Dhondt 1992; Lurz et al. 2000; Wauters et al. 2001, 2005; Romeo et al. 2010; Di Pierro et al. 2011). In most habitats, male squirrels have larger home ranges than females and overlap with several females to increase their probability of mating. In stable habitats, females tend to defend exclusive core-areas against other females and have smaller core areas than males (Wauters and Dhondt 1992; Lurz et al. 2000; Wauters et al. 2001; Di Pierro et al. 2008, 2011; Romeo et al. 2010). Hence, male and female red squirrels have different space use patterns. Females may copulate with more than one male, but the majority only mates with a dominant male of high body mass (Wauters and Dhondt 1989; Wauters et al. 1990). They can produce 1 to 2 litters/year, and reproductive success depends mainly on body condition and food availability (Wauters and Dhondt 1995; Wauters et al. 1995, 2007; Rodrigues et al. 2010).

### Study area and food availability

We studied red squirrels and counted cones produced yearly in three study areas in the Italian Alps in Northern Italy (Oga = OGA; Cedrasco = CED; Val di Rhêmes = RHE; Santicchia et al. 2018; details in Table ESM1). Annual estimates of conifer seed-crop size and the number of red squirrels used to estimate personality traits and space use are reported in Table ESM2. In this paper, we only used a categorical index of food abundance for each period in which squirrel home-range sizes were estimated: poor seed-crop versus medium–high seed-crop (Table ESM2).

### Trapping and handling

We trapped squirrels in three periods per year (April–May, June–July, September–October): from April 2000 to October 2006 in OGA, from April 2000 to April 2009 in CED and RHE. We used 25 (CED), 23 (OGA), or 30 (RHE) ground-placed Tomahawk traps (models 201 and 202, Tomahawk Live Trap Co., Hazelhurst, WI, USA), homogeneously distributed over the study areas (distance between traps 100–130 m; trap density 0.7–0.8 traps ha^−1^). Details on study area boundaries and edge effects are given in Santicchia et al. (2018). We pre-baited traps 4 times over a 30-day period using hazelnuts, then baited and activated for 6–10 days (Wauters et al. 2008). We checked traps three times per day. We marked each trapped squirrel using unique numbered metal ear-tags and weighed them using a spring-balance (± 5 g, Pesola AG, Baar, Switzerland). We determined sex and age class based on external genitalia and body mass (juveniles < 250 g; Wauters and Dhondt 1995; Wauters et al. 2007). See “Ethical note” for further details.

As in previous studies on tree squirrels (e.g. Kenward et al. 1998; Wauters et al. 2004, 2008; Boutin et al. 2006), we estimated population density, in each trapping period, using the minimum number of animals known to be alive (MNA) from CMR, radio-tracking and observations. Because of sex-specific space use and demographic processes, we calculated density for each sex separately (Wauters et al. 2004; Di Pierro et al. 2011).

### Radio-tracking

To study space use, since we aimed to estimate core-area overlap among squirrels, we radio-collared as many individuals as possible (no sample size restrictions). We radio-collared 36 adult red squirrels (22 males and 14 females) at CED, 22 adults (12 males, 10 females) at OGA, and 42 squirrels (23 males, 19 females) at RHE with species-specific collars. We used either PD-2C transmitters (8 g, < 4% of an individual’s body mass, Holohil Systems Ltd., Carp, Ontario, Canada) or TW-4 transmitters (12 g, < 5% of an individual’s body mass, Biotrack Ltd., Wareham, Dorset, UK) with adjustable necklace size. In all study areas, we took one or two locations per day (one during the morning activity bout, the second in the afternoon). The interval between consecutive radio-tracking days was irregular avoiding autocorrelation in location data. Of the 100 squirrels, 9 were predated (9%, below the average 20% of 6-month mortality-rate; from Wauters et al. 2004, 2008) and for 86 of the remaining 91 (95%) collars were removed.

We estimated locations (fixes) to the nearest 10 by 10 m by homing-in to the radio-signal (Wauters and Dhondt 1992; Wauters et al. 2001) and to estimate home-range and core-area size we used only squirrels for which we had between 23 and 45 fixes each. For each year, we estimated home ranges on a seasonal basis: spring–summer (April–July) and autumn (September–November). Since the space use of several individuals was monitored in different seasons and/or years, we had a total of 121 home-range and core-area size estimates of 64 different squirrels for which we also had personality data (males 73 estimates of 40 individuals, females 48 estimates of 24 individuals). We used the 95% fixed kernel probability density estimator with adjusted bandwidth *h* (KDE_adj_, Wauters et al. 2007, hereinafter KDE) to produced reliable estimates of home-range size (Di Pierro et al. 2008, 2011; Romeo et al. 2010). We estimated core-area size using the 85% Incremental Cluster Polygon (hereinafter core-area; see also Lurz et al. 2000; Wauters et al. 2005; Di Pierro et al. 2008, 2011) because the utilization distribution curve of core-area size on percentage of fixes used showed a clear inflection point between the 85 and 90% isopleths. Core-area overlap data were obtained from previous studies (Wauters et al. 2005; Romeo et al. 2010; Di Pierro et al. 2011). In summary, overlap of an individual’s core-area was calculated as the total % of overlap with the core-areas of all other radio-tracked squirrels. We calculated it for each sex separately rendering four combinations: a male by other males, a male by females, a female by males and a female by other females. Not all squirrels present in a given period were radio-collared (CED 80–100% of residents, OGA 75–77% of residents, RHE 60–87% of residents; from Wauters et al. 2005; Romeo et al. 2010; Di Pierro et al. 2011), resulting in a slight underestimation of core-area overlap inherent to most radio-telemetry studies. Radio-tracking data and home range analyses were described in Di Pierro et al. (2011) for CED, in Romeo et al. (2010) for OGA and in Wauters et al. (2005) for RHE.

Since space use, population density and body size (foot length and body mass) of squirrels differed among study areas (see also Wauters et al. 2005, 2007; Romeo et al. 2010), all continuous explanatory variables were standardised [*x*_*i*_ − mean *x*)/SD *x*] within each study area before using them in the LMM models that explored variation in space use determined by the animal’s personality, other intrinsic variables and environmental variables (see “Space use—personality models”).

### Ethical note

Our procedures of trapping, handling, marking and radio-tracking squirrels complied with the Guidelines for the treatment of animals in behavioural research and teaching (Animal Behaviour, 2020, 159, I–XI; 10.1016/j.anbehav.2019.11.002). We partly covered the Tomahawk Live Traps with a dark plastic bag to provide animals with shelter and checked traps three times/day to minimize the time in trap. Before handling, we completely covered the trap with a cloth to reduce stress. We flushed the trapped animal in a zipper-tube handling bag to reduce direct contact with the operator. At first capture, we marked each squirrel with a Monel 1005 1L1 ear-tag (size 2.3–10 mm, 0.2 g or less than 0.1% of squirrel’s body mass; National Band & Tag Co. Newport, KY, USA), putting the tag near the base of the ear to reduce risk of injury. There is no evidence that ear-tags affect grooming behaviour or the occurrence of ectoparasite around the ear region. To reduce stress, only trained researchers handled the squirrels, and handling time was kept as short as possible (< 5 min). The animals were released at the trap site immediately after handling. Since the study also aimed at estimating population size based on CMR, all animals captured were marked (no sample size restrictions).

Trapping and handling squirrels complied with the current laws on animal research in Italy and were carried out under the permission of the authorities for wildlife research and management of Lombardy Region and Gran Paradiso National Park. Legal requirements according to the Italian Wildlife Protection and Hunting Law L.N. 157 from 1992 and fieldwork was approved by authorization decrees n. 855 of 17/01/2000, n. 7489 of 29/04/2002, n. 10816 of 10/06/2002 and n. 1861 of 16/02/2004 from Direzione Generale Agricoltura, Regione Lombardia, Italy; and the permission (DGE25-2000) from the Gran Paradiso National Park, Italy.

### Personality: trappability and trap diversity indices

For each individual, we used the indices of trappability (number of captures/number of capture days from the first to the last trapping session an animal was present in the study area) and trap diversity (number of different traps in which an individual was captured/number of available traps in the study area). Trappability measures an animal’s tendency to take risks (boldness), while trap diversity measures willingness to explore novel environments (Boon et al. 2008; Boyer et al. 2010). Because the number of traps available and capture histories differed among study areas, the trappability and trap diversity indices were standardised within each area.

Since we analysed all space use—personality models for each sex separately and the repeatability of behaviours (i.e. within-individual consistency) may also vary in a sex-specific manner (Schuett and Dall 2009), we estimated the repeatability of trappability and trap diversity per sex on a subset of 44 males and 30 females trapped in more than one year. Since in this subset, the length of capture period and the number of available traps were constant over both years, we estimated the repeatability (*R*) in the number of captures per year and in trap diversity per year with a Linear Mixed Models (LMM) (Nakagawa and Schielzeth 2010). We used the R software (version 3.6.0) package rptR v 0.9.22 to estimate *R* and its 95% CIs (number of parametric bootstraps for interval estimation = 5000, number of permutations used when calculating asymptotic *P* values = 1000; see also Santicchia et al. 2018). We ln-transformed number of captures and square root transformed trap diversity (number of different traps) to meet assumptions of normality (Shapiro–Wilk’s test on transformed data, all *W* ≥ 0.94). We included study area, and year and their interactions as fixed effects and squirrel identity as random factor.

Finally, because standardised trappability and trap diversity were highly correlated (*r* = 0.82; *N* = 121; *P* < 0.0001), we used a Principal Component Analysis (PCA) to derive new non-correlated variables (see also Boyer et al. 2010; Santicchia et al. 2018, 2019). The loadings were PC1 = 0.707 * trappability + 0.707 * trap diversity; PC2 = 0.707 * trap diversity − 0.707 * trappability (Eigenvalues PC1 = 1.820, PC2 = 0.180). Since the first component explained 91% of the total variance in the PCA, we used only PC1 in our mixed models (see below). PC1 had a high score for bold squirrels with a strong exploration tendency, and a low score for shy, less explorative animals.

Trappability and trap diversity indices derived from standardised Capture-Mark-Recapture (CMR) studies have a moderate to good repeatability and represent reliable measures of the personality traits boldness and exploration in the habitat where the animal settled (Boon et al. 2008; Boyer et al. 2010; Le Coeur et al. 2015; Santicchia et al. 2018, 2019, 2020). We further refer to “Discussion” regarding possible potential caveats related to the use of these indices.

### Space use—personality models

Models that described broad patterns of variation in home-range and core-area size, and in the ratio of core-area/home-range are presented in ESM (ESM3 and Table ESM3). Since, as mentioned above, male and female red squirrels have different space use patterns (e.g. Wauters and Dhondt 1992; Lurz et al. 2000; Romeo et al. 2010), we analysed the effects of personality on space use for each sex separately (Santicchia et al. 2018).

We explored variation in space use using a LMM with standardised home-range or core-area size as the dependent variable, adding individual as a repeated measure to account for pseudoreplication (Verbeke and Molenberghs 2000). In the full model we used PC1 as an explanatory variable, and further included the squirrel’s body mass, density of animals of the same sex, density of animals of the other sex as continuous variable, and season and a food abundance index (low vs. medium–high) as categorical fixed effects. We tested whether space use—personality relationships were affected by changes in food abundance and/or population density (our predictions 4 and 5), by including the interactions of these variables with PC1. We did not use the number of fixes in the models because our earlier studies in these areas showed that variation in the number of fixes did not affect the space-use estimates after a threshold of 22 fixes was reached (Wauters et al. 2005; Romeo et al. 2010; Di Pierro et al. 2011).

We investigated which of three different correlation structures of the residual correlation matrix best fitted the data using Schwarz’s Bayesian Information Criterion (BIC), where lower values indicate better fit (Verbeke and Molenberghs 2000). We compared simple (no correlation between repeated measures on an individual), compound symmetry (CS; assuming a correlation between two measures on the same individual that does not vary over time) and first order autoregression correlation structures (assuming that the correlation between two measures on the same individual is a function of the time-interval between them). We used a stepwise backward model selection based on partial *p*-values eliminating non-significant interactions and fixed effects to produce selected models. Degrees of freedom and standard errors of *F*- and *t*-tests were obtained using Kenward–Rogers method (Verbeke and Molenberghs 2000). Model residuals did not deviate from a normal distribution (based on QQ-plots and Shapiro–Wilk’s statistic). The same LMM modelling was also used with the standardised ratio of core-area/home-range size as dependent variable.

Finally, we investigated the effects of personality on the patterns of core-area overlap, using the within study area standardised values of % core-area overlap as dependent variables. We modelled four different response variables: males overlap by other males, males overlap by females, females overlap by males and females overlap by other females (Wauters and Dhondt 1992; Romeo et al. 2010; Di Pierro et al. 2011). We tested the same fixed effects as in the models above, but we only considered density of the overlapping sex as the biologically relevant density measure; thus we included male density when overlap with males was modelled, female density when overlap with females was modelled. Model selection was carried out as described above.

All tests of significance are two-tailed and the significance level was set at 0.05. All the statistical analyses, except estimates of repeatability, were done using SAS/STAT 9.4 software (Copyright © 2011, SAS Institute Inc., Cary, NC, USA).

## Results

### Space use patterns

Individual variation in home-range and core-area size of red squirrels was large (*N* = 121; mean ± SD: KDE 23.45 ± 32.33 ha, range 1.26–194.20 ha; core-area 7.47 ± 10.13 ha, range 0.56–98.53 ha). The two space use estimators were positively correlated (*r* = 0.65; *N* = 121; *P* < 0.0001). Raw data of home-range and core-area size (in ha) per study area and sex are given in Table 1. Details on statistical analyses and differences between areas and the sexes can be found in ESM3 and Table ESM3.

**Table 1:** Red squirrel home-range size (mean ± SD) and core-area size (mean ± SD) variation between the sexes and among study areas

Study area	Sex (*N*)	95% KDE (ha)	Core area (ha)	Male ovl by (%)	Female ovl by (%)
CED	Males (27)	9.92 ± 12.72	4.30 ± 3.22	38 ± 45	26 ± 29
CED	Females (18)	6.11 ± 7.76	2.97 ± 3.66	61 ± 44	12 ± 21
OGA	Males (15)	9.83 ± 11.17	5.66 ± 5.17	42 ± 46	25 ± 32
OGA	Females (10)	3.88 ± 0.96	2.75 ± 0.66	51 ± 63	11 ± 14
RHE	Males (31)	44.54 ± 40.17	10.05 ± 6.18	50 ± 38	58 ± 48
RHE	Females (20)	40.61 ± 39.93	15.48 ± 20.38	49 ± 46	52 ± 66

Core-area overlap estimates (mean ± SD): male ovl by = average % overlap for a male by other males and by females (column Sex) in each study area; and female ovl by = average % overlap for a female by males and by other females (column Sex) in each study area

### Repeatability of trapping indices

Trappability and trap diversity indices were consistent through time and had a high repeatability (*R*) in both sexes (44 males: trappability *R* = 0.70, 95% CI = 0.54–0.84; trap diversity *R* = 0.64, 95% CI = 0.46–0.81; 30 females: trappability *R* = 0.65, 95% CI = 0.43–0.84; trap diversity *R* = 0.62, 95% CI = 0.39–0.83; all likelihood ratio test *P* < 0.001). Therefore, we consider them as suitable measures of, respectively, boldness and exploration in red squirrels. As explained above, because of their strong correlation, we performed a PCA on trappability and trap diversity and used the scores along the first axis (PC1 scores) as our final measure of personality in the space use models.

### Space use and personality

Full LMMs testing the effects of PC1 on each of the space use estimators (dependent variable) are given in Supplementary Material (Table ESM4 for males, Table ESM5 for females). An individual’s body mass and PC1 score were not correlated (males, *r* = 0.16; *N* = 73; *P* = 0.16; females *r* = 0.24; *N* = 48; *P* = 0.09).

Home-range and core-area size of male (*N* = 73 of 40 different animals) and female red squirrels (*N* = 48 of 24 different animals) were not affected by variation in personality (PC1) (Tables ESM4, ESM5). However, other factors affected their size. In males, home range size increased when food abundance was low (estimate low vs medium–high food = 0.69 ± 0.26; *t*_70_ = 2.68; *P* = 0.009), and decreased at high female density (estimate − 0.31 ± 0.16; *t*_70_ = 1.96; *P* = 0.054; Table ESM4a), while variation in core-area size was not affected by any of the fixed effects (Table ESM4b). In females, home-range size also tended to increase when female density decreased (selected model: female density effect estimate − 0.40 ± 0.14; *t*_42_ = 2.80; *P* = 0.008), and, as in males, variation in core-area size was not affected by any of the fixed effects (Table ESM5b).

**Fig. 1:**
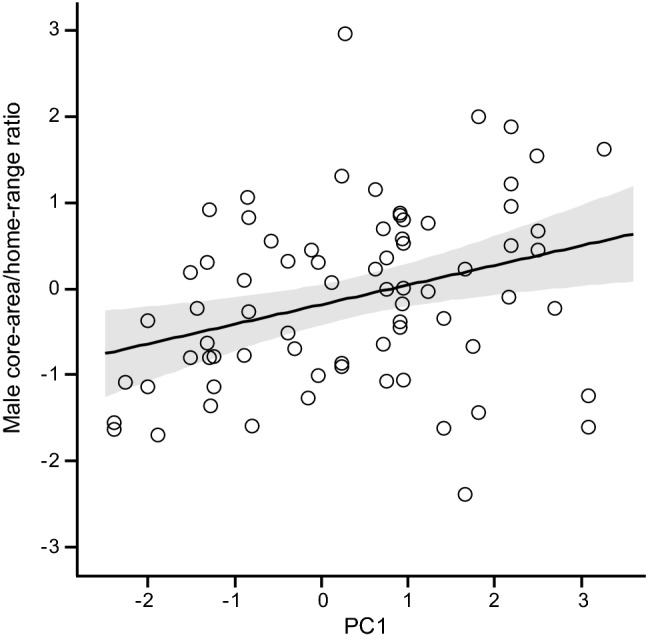
Relationship between core-area/home-range ratio and PC1 (standardised values) in male red squirrels (at density of females = − 0.5). The solid line represents the predicted relationship, shaded areas represent the 95% confidence interval, symbols represent observed values

We found a positive relationship of the standardised ratio of core-area/home-range with PC1 among male red squirrels (0.23 ± 0.08; *t*_70_ = 2.92; *P* = 0.0047), but not females. Thus, bolder and more explorative males (high PC1 score) used relatively larger core-areas within their home range than shy ones (Fig. 1). None of the other explanatory variables significantly affected variation in the standardised core-area/home-range ratio of males or females (Tables ESM4c, ESM5c).

**Fig. 2:**
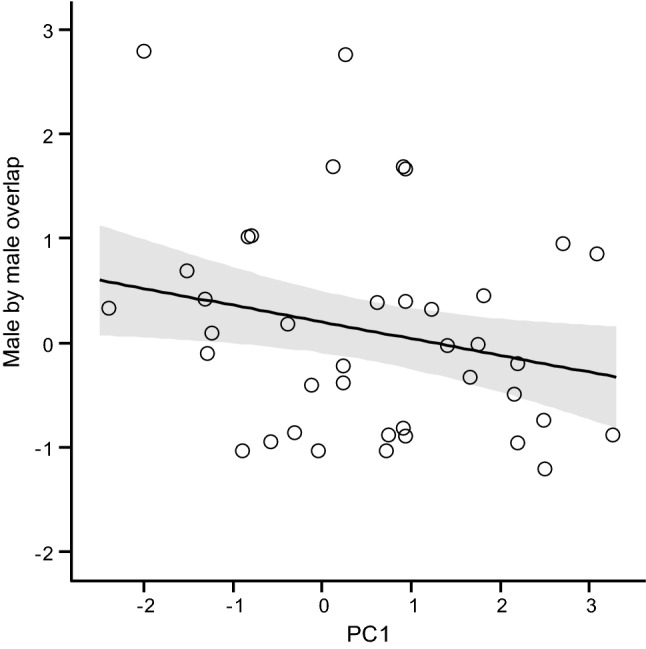
Relationship between core-area overlap among male red squirrels and PC1 (standardised values); season effect kept constant at spring season. The lines represent the predicted relationship, shaded areas represent the 95% confidence intervals and symbols represent observed values

Overlap of a male’s core-area with those of other males was higher in spring than in autumn (season effect 0.49 ± 0.21; *t*_70_ = 2.32; *P* = 0.023) and decreased with PC1 (− 0.16 ± 0.07; *t*_70_ = 2.21: *P* = 0.03; Fig. 2); hence bolder, more explorative males had less core-area overlap with other males than shy, less explorative ones (Table ESM6a). In the model of males overlapped by females, only food abundance had a significant effect: core-area overlap between a male and female squirrels increased at low food availability (food effect 0.64 ± 0.24; *t*_71_ = 2.69; *P* = 0.009; Table ESM6b). A female’s core-area overlap with males was not related to its personality and there was no effect of male density or food abundance on individual variation in female by male core-area overlap (Table ESM7a). The selected model of a female’s overlap by other females showed a significant effect of body mass (− 0.25 ± 0.12; *t*_46_ = 2.10; *P* = 0.041); heavier females had less intrasexual core-area overlap than those of lower body mass. A female’s personality did not affect the amount of intra-sexual overlap (Table ESM7b).

### Food- or density related associations between personality and space use

**Fig. 3:**
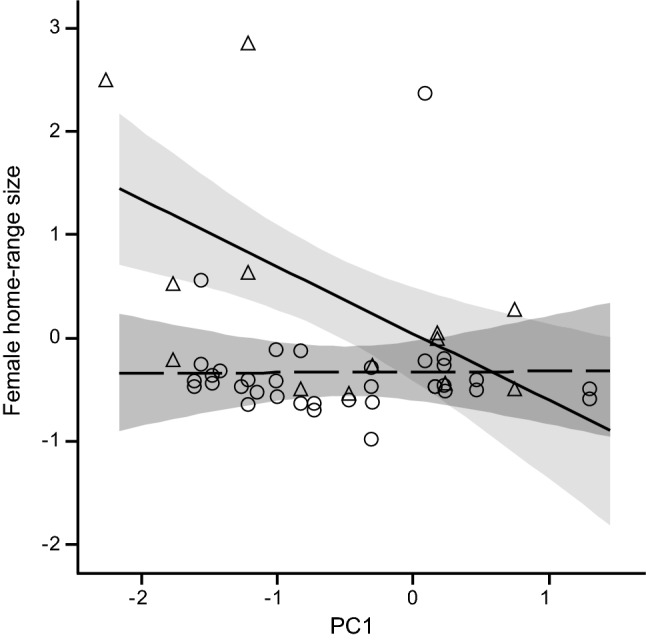
Relationship between home-range size and PC1 (standardised values) in female red squirrels at low (solid line, triangles) and medium–high (dashed line, circles) food availability (at density of females = − 0.66). The lines represent the predicted relationships, shaded areas represent the 95% confidence intervals, symbols represent observed values

We did not find any relationships between personality traits and spatial behaviour of male squirrels when environmental and/or social conditions fluctuated. Among female red squirrels the effect of personality (PC1) on home-range size differed with food availability (Fig. 3), and was negatively related with home-range size during poor seed-crops, but not so during periods with medium–high seed-crops (PC1 * food interaction − 0.61 ± 0.23; *t*_42_ = 2.71; *P* = 0.0098; Table ESM5a). Hence, there were no differences in home-range size between bold and shy females in years with medium to rich seed-crops, while bolder, more explorative females used smaller home ranges than shy, less explorative ones at low food availability (Fig. 3). We also found a nearly significant interaction of PC1 with female density (PC1 * Nfemales estimate − 0.30 ± 0.15; *t*_42_ = 1.97; *P* = 0.055). In response to fluctuations in female density, bolder females used larger home ranges than shy ones at low density, at medium densities, there was no effect of personality on home-range size, while at high female densities, bolder and more explorative animals tended to sue smaller home ranges than shy, less explorative ones.

## Discussion

Consistent differences in behaviour among individuals, hence animal personality, is a key candidate to determine inter-individual variation in space use and movements (Minderman et al. 2010; Spiegel et al. 2017; Schirmer et al. 2019; Wat et al. 2019). Furthermore, the personality traits that influence space use might differ between male and female vertebrates in relation to sex-biased strategies to maximize reproductive success (e.g. Le Coeur et al. 2015; Wat et al. 2019). Moreover, since spatio-temporal fluctuations in environmental variables, in particular food availability and density of conspecifics, will influence movement patterns, they are key candidates to interact with personality–spacing behaviour relationships. Whilst this is well established theoretically, we are among the first to have used multi-year and multi-site data to explore how the animal’s sex and changes in environmental factors interact with the complex relationships between space use and personality. Here, we found evidence for sex-specific effects of personality on home range size and space use patterns in populations of Eurasian red squirrels occupying different montane and subalpine conifer forests. Moreover, we showed that complex interactions of personality with seed-crop size and/or with sex-specific density influence a squirrels’ space use. In this study, we did not use standardized behavioural tests (e.g. arena tests), but adopted spatial capture-recapture data to assess whether repeatable, between-individual differences in behaviour exist. We analysed two indices of personality: trappability and trap diversity; however, since they were strongly correlated (see also Boyer et al. 2010; Santicchia et al. 2018, 2019), we used PCA to derive a single personality score. Hereinafter we shall refer to animals with high values along the first PCA axis (high PC1 score) as bolder squirrels that also tended to be the more explorative ones.

### Personality and space use: home range and core-area size

Contrary to our first prediction, personality traits were not directly related to absolute home-range or core-area size of male or female squirrels. Bolder, more explorative squirrels did not have larger home ranges or larger core-areas than shyer, less explorative ones. Variation in home-range size was mainly determined by access to limited resources, independent from the animal’s personality. Males increased their home range when food abundance and density of females (partners) were low, partly in agreement with our fourth prediction. Females increased their home-range size when female density decreased, confirming earlier studies showing that in this sex, intrasexual competition for space is the main driver of fluctuations in home-range size (Wauters and Dhondt 1992; Romeo et al. 2010).

### Personality and space structure: core-area/home-range ratio and core-area overlap

Personality influenced how males used the habitat inside their home range: bolder, more explorative males had a larger core-area/home-range ratio, and spatially their core-areas overlapped less with those of other males than for shy, less explorative ones. Hence, bolder and more explorative males intensively used a relatively large part of their total home range from which they are more prone to exclude other males, in agreement with our second and third prediction. This behaviour could result in an advantage to bolder males by increasing their access to limited food resources inside their core-areas. Similarly, bolder bank voles (*Myodes glareolus*) had smaller core-area overlap than shyer individuals. However, in contrast to male red squirrels, bolder voles also had larger home ranges and core-areas (Schirmer et al. 2019). In sleepy lizards (*Tiliqua rugosa*), core-area overlap was higher for unaggressive animals than for aggressive ones, while home-range size was positively associated with the behavioural trait boldness (Spiegel et al. 2015). The differences in male squirrels’ personality may also result in two different reproductive strategies. Bolder, more explorative males used relatively larger core-areas within their home range (core area/home range ratio) to increase access to food while shy, less explorative ones used relatively smaller core-areas. The latter suggests they might move over multinuclear core-areas that consist of several small but intensively used patches, a behaviour that could favour them when conifer seed availability is strongly reduced and alternative resources must be sought (Wauters et al. 2005). Finally, when female density was low, male squirrels used larger home ranges than at high female density, independently of their boldness-exploration level. Hence, all male red squirrels responded to fluctuations in the density of potential partners, which also explained higher overlap among males in spring–summer, when matings occur, compared to autumn.

Contrary to males, females’ space use within their home ranges (i.e. core-area/home-range ratio or overlap) did not vary with personality as predicted (prediction 2 and 3). However, intrasexual core-area overlap was inversely related to a female’s body mass, confirming the pattern of intrasexual territoriality among adult, dominant females of high body mass which is consistent over a wide range of habitats and densities (Wauters and Dhondt 1992; Lurz et al. 2000; Wauters et al. 2001; Romeo et al. 2010; Di Pierro et al. 2011). Hence, female red squirrels typically have low overlap with other females, independent of their personality.

### Personality, space use and changes in resources: food and squirrel density

Overall, both male and female red squirrels tended to increase their home-range size when food abundance was low and when female density in the population decreased; a pattern typical for this species (e.g. Wauters et al. 2001, 2005; Romeo et al. 2010; Di Pierro et al. 2011). Contrary to our first prediction, personality traits were not directly related to absolute home-range or core-area size of squirrels, however, in agreement with prediction 4, the relationship between individual personality and space use became relevant at specific extrinsic conditions, at least for females, with food availability being the most influential factor.

Among females, personality had no effect on home-range size at high food availability, but when food was scarce, bold-explorative females reduced home-range size whereas shy, less explorative individuals increased it. This counter-intuitive behaviour could be a reflection of habitat quality in that bold-explorative females have a better knowledge of and select high-quality patches that contained still sufficient food resources during low tree-seed availability. With medium–high seed-crops, female space use was more stable and independent from boldness-exploration tendency. This pattern suggests that bold, explorative females are more aware of their surroundings and the distribution and availability of food resources than shy, less explorative ones. Spatial knowledge about the changing distribution of food resources is key, and these females are therefore quicker to respond to periods of poor-seed-crops by shifting their home range to the few high-quality habitat patches that allow a relatively high daily energy-intake, resulting in smaller ranges. In contrast, shy and less explorative individuals will be forced to increase the size of their foraging grounds to meet their energy requirements. This is likely an adaptive strategy, as our study areas were characterised by high spatio-temporal variation in the abundance of conifer seeds, the squirrels’ main food supply (Wauters et al. 2005; Romeo et al. 2010; Di Pierro et al. 2011; and Table ESM2). Food availability did not only fluctuate annually, but there was considerable spatial variation, which was more extreme in years of poor seed-crops (coefficient of variation of seed-crop estimates over the 20 sampling plots: poor food years 91 to 221%, average 150%; medium–high food years 38 to 105%, average 68%; from Table ESM2). Thus, when food availability is medium–high, most trees produce cones and spatial distribution of food resources is more homogeneous than with low food.

A similar sex-specific pattern was found in the common brushtail possum (*Trichosurus vulpecula*) where less explorative females, yet high explorative males, had larger home ranges (Wat et al. 2019). Also, in a study with juvenile great tits (*Parus major*) fast explorers rapidly shifted to different foraging areas, but did not show a larger increase in home-range size than slow explorers when the food supply was experimentally reduced (van Overveld and Matthysen 2010). These authors suggested that slow and fast explorers differed in how they used the information collected on temporal changes in food availability, but not in the extent of space used for foraging (van Overveld and Matthysen 2010). In contrast in starlings (*Sturnus vulgaris*), the relationship between an exploration score and home-rage size was positive, but it also became more evident when local density (flock size) was high and habitat quality low (Minderman et al. 2010).

Finally, we found a weak, and not-significant tendency for an interaction of PC1 with female density on fluctuations in home-range size. Shy and less explorative females (low PC1 score) did not vary their home-range size with fluctuating female densities, while bolder and more explorative females tended to use larger ranges than shy, less explorative ones at low densities, while at high densities the trend was opposite. Tentatively, this suggests that bolder, more explorative females seemed to be more flexible in response to intra-sexual competition, which could enhance their access to higher-quality foraging patches (e.g. Patrick and Weimerskirch 2014).

### Potential caveats of the study methods

The reliability of trappability and trap diversity indices as proxies of, respectively, boldness and exploration in red squirrels have been discussed by Santicchia et al. (2018). Nevertheless, one might argue that trap diversity, which we used as our measure of adult squirrels’ exploration in a known environment, is a proxy of home-range or core-area size rather than a personality index, since home-range size affects how many traps the owner can potentially visit. We are convinced this is not the case because: (1) trap diversity had a high repeatability, suggesting it indeed measures a personality trait; and more importantly; (2) there was no positive correlation between home-range (Pearson correlation test *r* = − 0.13) or core area size (*r* = − 0.11) and trap diversity; (3) as described in the methods, the periods over which trappability and trap diversity were estimated did not overlap strongly with the (generally shorter) periods of radio-tracking. Most animals were trapped both before, during and after space use parameters were determined and some traps in which they were caught were outside the estimated home range area (Santicchia et al. 2018). Moreover, we are confident that our standardised methods of pre-baiting traps and seasonally spaced multiple days CMR sessions strongly reduced any potential bias in trappability [see also Michelangeli et al. 2016 in delicate skink (*Lampropholis delicata*); Jolly et al. 2019 in grassland melomys (*Melomys burtoni*)] as discussed in detail in previous studies on red squirrels (Wauters et al. 2008; Santicchia et al. 2018, 2020). Future research with the use of GPS-collars, which provide continuous animals’ locations and complete movements recordings (e.g. Melovski et al. 2020; Pisanu et al. 2020), will allow corroborating these assumptions.

## Conclusions

Male and female red squirrels adapted their space use in different ways to fluctuations in squirrel density and/or food abundance. Moreover, inter-individual variation in red squirrel space use was, to some extend, influenced by their personality, but these relationships were highly context-driven (see also Minderman et al. 2010; Dingemanse and Wolf 2010) and differed with sex. For example, in males, there was a direct effect of boldness, exploration tendency on home-range use (measured with core-area/home-range ratio), while among females, bolder, more explorative females reduced their home ranges when food availability was low, but there was no effect of personality on home-range size at medium–high seed-crops. Hence, the capacity to acquire information about changing environmental variables (e.g. food resources, competitors, partners) is likely to differ between the various personality types, which will feedback to their movement and space use decisions (Spiegel and Crofoot 2016). Thus, different space use strategies between the sexes to maximize access to limited, and seasonally changing resources (food resources for females, partners and food resources for males), linked to differences in personality, resulted in individual variation in home-range size and space use in populations. Variation in spacing behaviour and changing fitness advantages (e.g. Le Coeur et al. 2015; Santicchia et al. 2018) of animals with different personalities will further enhance the possibility that at least part of the population will respond successfully to strong fluctuations in resource abundance in boom and bust production-consumer systems, guaranteeing the long-term persistence of the populations.

## Supplementary Information

Below is the link to the electronic supplementary material.

Supplementary file 1 (DOCX 37 KB)

Supplementary file 2 (XLSX 31 KB)

## Data Availability

The datafile is added in Electronic Supplementary Material 2 (ESM2: Wauters-etal-datafile.xlsx).
