# Supplementary material for: Personality traits, sex and food abundance shape space use in an arboreal mammal
Source: Oecologia. 2021 Apr 1;196(1):65–76. doi: 10.1007/s00442-021-04901-2 (PMC8139925; doi:10.1007/s00442-021-04901-2)
Supplement: Supplementary file 1 — (DOCX 37 KB) [file 442_2021_4901_MOESM1_ESM.docx]

**Electronic Supplementary Material (ESM)**

**Title: Personality traits, sex and food abundance shape space use in an arboreal mammal**

**Journal: Oecologia**

Authors: Wauters LA^a, b, †^; Mazzamuto MV^a, c, †^, Santicchia F^a^**^*^**, Martinoli A^a^, Preatoni DG^a^, Lurz PWW^d^ , Bertolino S^e^, Romeo C^f^

Corresponding author and affiliation

Francesca Santicchia, Environment Analysis and Management Unit - Guido Tosi Research Group - Department of Theoretical and Applied Sciences, Università degli Studi dell’Insubria, Varese, Italy

Email: f.santicchia@uninsubria.it

**Table ESM1.** Study areas and tree-species composition (% cover for each species). Area size and mean tree density ± SD reported under the name of the area. Distances between the areas are reported in Trizio et al. (2005). Cedrasco (CED, 46°07’ N, 9°48’ E, elevation 1100 – 1600 m a.s.l.) and Oga (OGA, 46º28´N, 10º22´E, elevation from 1280 m to 1450 m a.s.l.) are in Lombardy, while Val di Rhêmes (RHE, 45°39’N, 7°12’E, elevation 1740 – 1890 m a.s.l.) is located in the Gran Paradiso National Park in Aosta valley. CED and OGA are mixed montane conifer forests, while RHE is a mature Norway spruce forest.

|  | **CED** | **OGA** | **RHE** |
| --- | --- | --- | --- |
|  | 76 ha  449 ± 163 trees/ha | 47 ha  765 ± 251 trees/ha | 69 ha  773 ± 333 trees/ha |
| Silver fir (*Abies alba*) | 56 | - | - |
| Norway spruce (*Picea abies*) | 25 | 8.7 | 85 |
| Scots pine (*Pinus sylvestris*) | 5 | 88.5 | - |
| Larch (*Larix decidua*) | 3 | 2.5 | 11 |
| dead trees | 5 | 0.3 | 2 |
| Other | 6 | 0 | 2 |

**Table ESM2.** Food abundance estimates (conifer seed-crop in 10^3^ kJ/ha, mean ± SE) and number of space-use estimates (including repeated measures of the same animal) of radio-tracked red squirrels (males, females) for which also personality measures were available. Data per study area and year (from Romeo et al. 2010; Di Pierro et al. 2011; Santicchia et al. 2018).

| Year | Study areas |  |  |  |  |  |
| --- | --- | --- | --- | --- | --- | --- |
|  | CED |  | OGA |  | RHE |  |
|  | Food | Squirrels | Food | Squirrels | Food | Squirrels |
| 1999 | 4357 ± 570 |  | 1732 ± 419 |  |  |  |
| 2000 | 159 ± 63 * | 1 (1, 0) | 391 ± 82 * | 9 (7, 2) | 497 ± 252 * |  |
| 2001 | 3087 ± 440 |  | 588 ± 83 |  | 913 ± 109 | 23 (14, 9) |
| 2002 | 1867 ± 296 | 14 (9, 5) | 735 ± 153 |  | 1773 ± 154 | 14 (7, 5) |
| 2003 | 846 ± 226 * |  | 552 ± 81 | 16 (8, 8) | 1010 ± 130 | 18 (10, 6) |
| 2004 | 6142 ± 728 | 18 (10, 8) | 863 ± 111 |  | 2402 ± 233 |  |
| 2005 | 4022 ± 828 |  | 352 ± 73 * |  | 22 ± 5 * |  |
| 2006 | 6249 ± 907 | 12 (7, 5) |  |  | 1363 ± 179 |  |
| Total |  | 45 (27, 18) |  | 25 (15, 10) |  | 51 (31, 20) |

*years of poor seed-crop

**ESM3. LMM model on broad patterns of red squirrel space use**

In a preliminary step we analysed broad patterns of variation in home-range and core-area size, and in the ratio of core-area/home-range, with a LMM where the dependent variable was KDE and core-area (Ln transformed), and the core-area/home-range ratio (85%ICP on 95%KDE ratio) (one model for each space use estimator). We tested for the fixed effects of study area, sex and season, and for a sex*area and sex*season interaction. We added year as random factor and individual as a repeated measure to account for pseudoreplication (Verbeke and Molenberghs 2000). Degrees of freedom and standard errors of F- and t-tests were obtained using Kenward-Rogers method (Verbeke and Molenberghs 2000). We compared three different correlation structures of the residual correlation matrix (simple, corresponding to the absence of an effect of repeated measures on an individual, CS = compound symmetry, assuming a correlation between two measures on the same individual that does not vary over time, AR(1) = first order autoregression assuming that the correlation between two measures on the same individual is a function of the time-interval between them, Verbeke and Molenberghs 2000). In all cases, models with simple correlation structure had the smaller values of Schwarz’s Bayesian Information Criterion (BIC), indicating better fit (Verbeke and Molenberghs 2000). Model residuals did not deviate from a normal distribution (based on QQ-plots and Shapiro-Wilk’s statistic). Using stepwise backward model selection, the sex*season interaction was not significant in any of the three models and eliminated (sex*season: all partial *P* > 0.50).

**Table ESM3.** Three LMMs each with one space use estimator as dependent variable [core-area/home-range (CA/HR) ratio; Ln KDE; Ln core-area; see methods). Fixed effects of study area, sex, season and the sex by study area interaction (see ESM3 for full model and model selection details).

| Dependent var | CA/HR ratio | | Ln KDE |  | Ln core-area |  |
| --- | --- | --- | --- | --- | --- | --- |
| Fixed effects | *F* | *P* | *F* | *P* | *F* | *P* |
| Study area | *F*_2, 44_ = 8.76 | 0.0006 | *F*_2, 107_ =30.7 | <0.0001 | *F*_2, 34_ = 19.6 | <0.0001 |
| Sex | *F*_1, 111_ = 2.01 | 0.16 | *F*_1, 109_ = 5.85 | 0.017 | *F*_1, 111_ = 2.17 | 0.14 |
| Season | *F*_1, 108_ = 0.86 | 0.36 | *F*_1, 109_ = 2.03 | 0.16 | *F*_1, 108_ = 0.09 | 0.77 |
| Sex*area | *F*_2, 110_ = 0.48 | 0.62 | *F*_2, 109_ = 2.10 | 0.13 | *F*_2, 110_ = 3.77 | 0.026 |

Red squirrels had larger home ranges (Ln KDE) in RHE than in CED and OGA (pairwise comparisons, both p < 0.0001) and males tended to use larger home ranges than females (Table S3). However, in RHE there was no difference in home-range size between the sexes (Table S3; DLSM males – females: CED 0.41 ± 0.19, *P* = 0.03; OGA 0.50 ± 0.26, *P* = 0.054; RHE -0.03 ± 0.18, *P* = 0.87). Also core-area size differed among study areas (Table 1 and Table S3), with squirrels using larger core-areas in RHE than in CED and OGA (pairwise comparisons, both *P* < 0.0001). Only in CED males had larger core-areas than females (DLSM CED 0.44 ± 0.19, *P* = 0.022; OGA 0.34 ± 0.26, *P* = 0.20; RHE -0.23 ± 0.18, *P* = 0.19). The core-area/home-range ratio differed among the study areas, but not between the sexes or seasons (Table 1 and Table S3). It was higher in CED and OGA than in RHE (DLSM CED-RHE 0.13 ± 0.06, *P* = 0.05; OGA-RHE 0.24 ± 0.06, *P* = 0.0001).

**Table ESM4.** LMM (full model details, see methods) testing the effects of the Principal Component PC1 as a personality index on space use of male red squirrels. Season effect estimates spring versus autumn; food effect estimates poor seed-crop versus medium to good seed-crop. a) standardised 95%KDE as dependent variable; b) standardised 85% Core-area as dependent variable; c) Core-area/home-range ratio as dependent variable.

1. 95%KDE. Simple correlation structure had lowest BIC (229.0). Statistical inference based on partial *P*-values.

| Eliminated parameters |  | *t*-value | df | *P* |
| --- | --- | --- | --- | --- |
| PC1*food |  | 0.12 | 63 | 0.90 |
| Season |  | 0.33 | 64 | 0.74 |
| Body mass |  | 0.51 | 65 | 0.61 |
| PC1*Nmales |  | 1.21 | 66 | 0.23 |
| Nmales |  | 0.28 | 67 | 0.78 |
| PC1*Nfemales |  | 1.34 | 68 | 0.19 |
| PC1 |  | 0.89 | 69 | 0.38 |
| Selected model | Estimate ± SE |  |  |  |
| Nfemales | -0.31 ± 0.16 | 1.97 | 70 | 0.054 |
| Food | 0.69 ± 0.26 | 2.68 | 70 | 0.009 |

1. 85% ICP core-area. Simple correlation structure had lowest BIC (206.9). Statistical inference based on partial *P*-values.

| Eliminated Parameters |  | *t*-value | df | *P* |
| --- | --- | --- | --- | --- |
| Season |  | 0.16 | 63 | 0.87 |
| Body mass |  | 0.21 | 64 | 0.84 |
| PC1*food |  | 0.19 | 65 | 0.85 |
| PC1*Nmales |  | 0.21 | 66 | 0.84 |
| PC1*Nfemales |  | 0.47 | 67 | 0.64 |
| Nfemales |  | 0.13 | 68 | 0.90 |
| Nmales |  | 0.20 | 69 | 0.84 |
| PC1 |  | 1.03 | 70 | 0.31 |
|  | Estimate ± SE |  |  |  |
| Food | 0.25 ± 0.21 | 1.18 | 71 | 0.24 |

1. CA/HR ratio. Simple correlation structure had lowest BIC (217.8). Statistical inference based on partial *P*-values.

| Parameter |  | *t*-value | df | *P* |
| --- | --- | --- | --- | --- |
| PC1*food |  | 0.23 | 63 | 0.82 |
| Season |  | 0.10 | 64 | 0.92 |
| PC1*Nmales |  | 0.83 | 65 | 0.41 |
| Nmales |  | 0.72 | 66 | 0.47 |
| Body mass |  | 1.24 | 67 | 0.22 |
| PC1*Nfemales |  | 1.53 | 68 | 0.13 |
| Food |  | 1.43 | 69 | 0.16 |
| Selected model | Estimate ± SE |  |  |  |
| Nfemales | 0.25 ± 0.15 | 1.75 | 70 | 0.085 |
| PC1 | 0.23 ± 0.08 | 2.92 | 70 | 0.0047 |

**Table ESM5.** LMM (full model details, see methods) testing the effects of the Principal Component PC1 as a personality index on space use of female red squirrels. Season effect estimates spring versus autumn; food effect estimates poor seed-crop versus medium to good seed-crop. a) standardised 95%KDE as dependent variable; b) standardised 85% Core-area as dependent variable; c) Core-area/home-range ratio as dependent variable.

1. 95%KDE. Simple correlation structure had lowest BIC (BIC = 102.2). Statistical inference based on partial *P*-values.

| Parameter |  | *t*-value | df | *P* |
| --- | --- | --- | --- | --- |
| Season |  | 0.33 | 38 | 0.74 |
| PC1*Nmales |  | 1.64 | 39 | 0.11 |
| Body mass |  | 1.88 | 40 | 0.067 |
| Nmales |  | 1.43 | 41 | 0.16 |
| Selected model | Estimate ± SE |  |  |  |
| PC1^b^ |  | 1.12 | 42 | 0.27 |
| Nfemales |  | 2.80 | 42 | 0.008 |
| PC1*Nfemales | -0.30 ± 0.15 | 1.97 | 42 | 0.055 |
| Food |  | 1.56 | 42 | 0.13 |
| Pc1*food^a^ | -0.61 ± 0.35 | 2.71 | 42 | 0.0098 |

^a^ Food medium-high as reference value

^b^ *F*-test on PC1 *F*_1, 42_ = 9.28; *P* = 0.004

1. 85% ICP core-area. Simple correlation structure had lowest BIC (BIC = 150.6). Statistical inference based on partial *P*-values.

| Parameter |  | *t*-value | df | *P* |
| --- | --- | --- | --- | --- |
| Season |  | 0.39 | 38 | 0.70 |
| Body mass |  | 0.27 | 39 | 0.79 |
| PC1*Nmales |  | 1.02 | 40 | 0.31 |
| Nmales |  | 0.96 | 41 | 0.34 |
| PC1*Nfemales |  | 1.62 | 42 | 0.11 |
| Nfemales |  | 1.34 | 43 | 0.19 |
| PC1*food | -0.69 ± 0.41 | 1.71 | 44 | 0.094 |
| PC1 |  | 0.39 | 45 | 0.70 |
| Selected model | Estimate ± SE |  |  |  |
| Food | 0.67± 0.37 | 1.80 | 46 | 0.078 |

1. CA/HR ratio. Simple correlation structure had lowest BIC (BIC = 138.1). Statistical inference based on partial *P*-values.

| Parameter |  | *t*-value | df | *P* |
| --- | --- | --- | --- | --- |
| PC1*Nfemales |  | 0.07 | 38 | 0.94 |
| Nfemales |  | 0.39 | 39 | 0.70 |
| PC1*Nmales |  | 0.23 | 40 | 0.82 |
| Nmales |  | 0.40 | 41 | 0.69 |
| PC1*food |  | 0.49 | 42 | 0.63 |
| Season |  | 0.84 | 43 | 0.41 |
| PC1 |  | 0.64 | 44 | 0.53 |
| Food |  | 1.11 | 45 | 0.27 |
| Body mass |  | 1.47 | 46 | 0.15 |

**Table ESM6.** LMM (full model details, see methods) testing the effects of trappability and trap diversity indices on core-area overlap in male red squirrels. Season effect estimate spring versus autumn; Food effect estimate poor seed-crop versus medium to good seed-crop. a) standardised overlap with other males as dependent variable; b) standardised overlap with females as dependent variable.

1. M by M overlap. Simple correlation structure had lowest BIC (BIC = 206.8). Statistical inference based on partial *P*-values.

| Eliminated parameters |  | *t*-value | df | *P* |
| --- | --- | --- | --- | --- |
| PC1*food |  | 0.36 | 65 | 0.72 |
| PC1*Nmales |  | 0.73 | 66 | 0.47 |
| Nmales |  | 0.06 | 67 | 0.95 |
| Food |  | 0.30 | 68 | 0.76 |
| Body mass |  | 1.21 | 69 | 0.23 |
| Selected model | Estimate ± SE |  |  |  |
| Season | 0.49 ± 0.21 | 2.32 | 70 | 0.023 |
| PC1 | -0.16 ± 0.07 | 2.21 | 70 | 0.030 |

1. M by F overlap. Simple correlation structure had lowest BIC (BIC = 211.0). Statistical inference based on partial *P*-values.

| Eliminated parameters |  | *t*-value | df | *P* |
| --- | --- | --- | --- | --- |
| PC1*food |  | 0.88 | 65 | 0.38 |
| Body mass |  | 1.29 | 66 | 0.20 |
| PC1*Nfemales |  | 1.59 | 67 | 0.12 |
| Nfemales |  | 0.14 | 68 | 0.89 |
| Season |  | 1.49 | 69 | 0.14 |
| PC1 |  | 1.03 | 70 | 0.31 |
| Selected model | Estimate ± SE |  |  |  |
| Food | 0.64 ± 0.24 | 2.69 | 71 | 0.009 |

**Table ESM7.** LMM (full model details, see methods) testing the effects of trappability and trap diversity indices on core-area overlap in female red squirrels. Season effect estimate spring versus autumn; Food effect estimate poor seed-crop versus medium to good seed-crop. a) standardised overlap with males as dependent variable; b) standardised overlap with other females as dependent variable.

1. F by M overlap. Simple correlation structure had lowest BIC (BIC = 147.5). Statistical inference based on partial *P*-values.

| Eliminated parameters |  | *t*-value | df | *P* |
| --- | --- | --- | --- | --- |
| PC1*Nmales |  | 0.33 | 40 | 0.75 |
| Body mass |  | 0.65 | 41 | 0.52 |
| Season |  | 0.64 | 42 | 0.53 |
| PC1*food |  | 0.69 | 43 | 0.49 |
| Nmales |  | 1.08 | 44 | 0.29 |
| Food |  | 1.51 | 45 | 0.14 |
| Selected model | Estimate ± SE |  |  |  |
| PC1 | -0.14 ± 0.18 | 0.78 | 46 | 0.44 |

1. F by F overlap. Simple correlation structure had lowest BIC (BIC = 135.9). Statistical inference based on partial *P*-values.

| Eliminated parameters |  | *t*-value | df | *P* |
| --- | --- | --- | --- | --- |
| PC1*food |  | 0.07 | 40 | 0.95 |
| PC1*Nfemales |  | 0.17 | 41 | 0.87 |
| Nfemales |  | 0.31 | 42 | 0.76 |
| Season |  | 1.13 | 43 | 0.27 |
| PC1 |  | 0.27 | 44 | 0.79 |
| Food |  | 1.10 | 45 | 0.28 |
| Selected model | Estimate ± SE |  |  |  |
| Body mass | -0.25 ± 0.12 | 2.10 | 46 | 0.041 |

**References ESM**

Di Pierro E, Ghisla A, Wauters LA, Molinari A, Martinoli A, Gurnell J, Tosi G (2011) The effects of seed availability on habitat use by a specialist seed predator. European Journal of Wildlife Research 57:585–595. <https://doi.org/10.1007/s10344-010-0469-7>

Romeo C, Wauters L, Preatoni D, Tosi G, Martinoli A (2010) Living on the edge: space use of Eurasian red squirrels in marginal high elevation habitat. Acta Oecologica 36:604–610. <https://doi.org/10.1016/j.actao.2010.09.005>

Santicchia F, Gagnaison C, Bisi F, Martinoli A, Matthysen E, Bertolino S, Wauters LA (2018) Habitat-dependent effects of personality on survival and reproduction in red squirrels. Behavioral Ecology and Sociobiology 72:134. <https://doi.org/10.1007/s00265-018-2546-y>

Trizio I, Crestanello B, Galbusera P, Wauters LA, Tosi G, Matthysen E, Hauffe HC (2005) Geographical distance and physical barriers shape the genetic structure of Eurasian red squirrels in the Italian alps. Molecular Ecology 14:469–481. <https://doi.org/10.1111/j.1365-294X.2005.02428.x>

Verbeke G and Molenberghs G. Linear mixed models for longitudinal data. New York: Springer, 2000
